# Association between multimorbidity patterns and chronic pain in elderly primary care patients: a cross-sectional observational study

**DOI:** 10.1186/s12875-016-0468-1

**Published:** 2016-06-06

**Authors:** Martin Scherer, Heike Hansen, Jochen Gensichen, Karola Mergenthal, Steffi Riedel-Heller, Siegfried Weyerer, Wolfgang Maier, Angela Fuchs, Horst Bickel, Gerhard Schön, Birgitt Wiese, Hans-Helmut König, Hendrik van den Bussche, Ingmar Schäfer

**Affiliations:** Department of Primary Medical Care, University Medical Center Hamburg-Eppendorf, Hamburg, Germany; Institute of General Practice, University of Jena, Jena, Germany; Institute of General Practice, University of Frankfurt am Main, Frankfurt am Main, Germany; Institute for Social Medicine, Occupational Health and Public Health, University of Leipzig, Leipzig, Germany; Central Institute of Mental Health, University of Heidelberg, Mannheim, Germany; Department of Psychiatry and Psychotherapy, University of Bonn, Bonn, Germany; Institute of General Practice, University of Düsseldorf, Düsseldorf, Germany; Department of Psychiatry and Psychotherapy, Technical University of Munich, Munich, Germany; Department of Medical Biometry and Epidemiology, University Medical Center Hamburg-Eppendorf, Hamburg, Germany; Institute for General Practice, Hannover Medical School, Hannover, Germany; Department of Health Economics and Health Services Research, University Medical Center Hamburg-Eppendorf, Hamburg, Germany

**Keywords:** Multimorbidity, Chronic pain, Low back pain, Patient-reported outcomes, CART analysis, Exporative analysis, Epidemiology

## Abstract

**Background:**

Multimorbidity is a highly prevalent health problem, which may reduce adherence, produce conflicts in treatment, and is not yet supported by evidence-based clinical recommendations. Many older people suffer from more than one chronic disease as well as from chronic pain. There is some evidence that disease management can become more complex if multimorbid patients suffer from chronic pain. In order to better consider the patients’ comorbidity spectrum in clinical pain treatment recommendations, evidence is needed regarding which disease combinations are frequently related with the presence of chronic pain. Therefore, our aim is to identify diseases and disease combinations in a multimorbid population, which are associated with the patient-reported presence of chronic pain.

**Methods:**

Analyses are based on cross-sectional data of the MultiCare Cohort Study, an observational cohort study based on interviews with 3189 multimorbid patients aged 65+, randomly selected from 158 practices, and their GPs. The response rate was 46.2 %. Data were collected in GP interviews and comprehensive patient interviews. Diseases and disease combinations associated with chronic pain were identified by CART (classification and regression tree) analyses performed separately for both genders. 46 chronic conditions were used as predictor variables and a dichotomized score from the Graded Chronic Pain Scale was used as outcome variable.

**Results:**

About 60 % of the study participants were female. Women more often reported chronic pain than men. The most important predictor of a higher pain level in the female population was chronic low back problems, especially if combined with chronic gastritis, hyperuricemia/gout, cardiac insufficiency, neuropathies or depression. Regarding the pain level the male population was also divided best by chronic low back problems, especially if combined with intestinal diverticulosis, neuropathies or chronic ischemic heart disease.

**Conclusions:**

Our analyses are a first step in identifying diseases and disease combinations that are related to chronic pain. The most important condition seems to be low back problems. Back pain and pain in other body regions seems to be interrelated with cardiometabolic conditions. In women, psychosocial issues like depression also seem to be relevant.

**Trial registration:**

ISRCTN89818205.

## Background

Multimorbidity is a highly prevalent health problem. Depending on definition and operationalization studies show prevalence rates in the elderly ranging from 55 to 98 % [1]. For the phenomenon of multimorbidity no uniform definition exists [2, 3]. We defined multimorbidity as the coexistence of at least three chronic diseases [4]. There are two different approaches to operationalize multimorbidity. The first approach is based on the assumption that not the effects of the individual diseases but rather their combined impact are relevant for the patient [5, 6]. However, in multimorbidity research a generalization of multimorbidity (e.g., though using a disease count) will probably underestimate the influence of multimorbidity, because chronic diseases are very heterogeneous regarding their effects and a generalized measure probably evens out the impact of the single diseases. For this reason we regard the second approach to multimorbidity as more promising. This approach assumes that health outcomes in multimorbid patients are mainly influenced by the pathophysiology of the single diseases [7], but that there may be additional effects of disease interactions. We therefore operationalized multimorbidity through diseases and disease combinations and evaluated their individual effect on the patient.

Multimorbidity is associated with many adverse patient-related health outcomes like decline of functional status, lower quality of life, higher mortality, increased health care utilization and therefore rising costs of care [1, 8]. A large number of multimorbid patients suffer from pain-related morbidity like chronic back problems, osteoarthritis, migraine or chronic gastritis [9]. However, the question of how and to what extent multimorbidity is interrelated with chronic pain has rarely been examined. Chronic pain can be the result of a multitude of different health problems. The International Association for the Study of Pain defines chronic pain as pain which persists beyond normal tissue healing time. According to a systematic review by Wolff et al. [10] about 17 % of the German population suffer from chronic pain. Many older people suffer from multimorbidity as well as from chronic pain [11] and about one third of elderly patients with multimorbidity take analgesics regularly or as needed [12].

Care for patients with multimorbidity is a challenging task for the GP because of three reasons: Firstly, multimorbidity may produce conflicts in treatment decisions, because indications and contra-indications for a specific treatment are often found in the same person. Secondly, multimorbidity is not yet supported by evidence-based clinical recommendations. Thirdly, due to its complexity, multimorbidity may reduce patients’ adherence to treatment plans [13]. The influence of chronic pain on the treatment of multimorbidity is not well understood, yet. However, there is some evidence that disease management can become more complex if multimorbid patients suffer from chronic pain, because dosing and adverse effect profiles of pain medication can be affected by other diseases present in the same person [14]. At the same time chronic pain can cause difficulties in performing essential self-mananagement activities of diseases like diabetes mellitus [11]. In order to better consider the patients’ comorbidity spectrum in clinical pain treatment recommendations, evidence is needed regarding which disease combinations are frequently related with the presence of chronic pain. The aim of this study therefore is to identify diseases and disease combinations in multimorbid patients which are associated with the patient-reported presence of chronic pain.

## Methods

### Study design and sample

The analyses are based on baseline data from the MultiCare Cohort Study (Trial registration ISRCTN89818205). The methods of this study have been described in detail in the published study protocol [4]. In short, it is designed as a multicenter, prospective, observational cohort study of multimorbid patients from general practice. The patients were recruited from 158 general practitioner (GP) practices in 8 major cities distributed across Germany (Bonn, Düsseldorf, Frankfurt/Main, Hamburg, Jena, Leipzig, Mannheim and Munich). In each practice we created a list of all patients from this practice who were born between 1.7.1923 and 30.6.1943 (i.e., between 65 and 85 years old) and consulted the GP at least once within the last completed quarter (i.e., 3 month period). Per practice, we randomly selected 50 patients with multimorbidity from this list and contacted them for written informed consent. Multimorbidity was defined as coexistence of at least three chronic conditions out of a list of 29 diseases.

Patients were excluded from the study if they were no regular patients of the participating practice (i.e., in case of accidental consultation of the GP), if they were unable to participate in interviews (especially blindness and deafness) or if they were not able to speak and read German. Further exclusion criteria were residence in a nursing home, severe illness probably lethal within three months according to the GP, insufficient ability to consent (especially dementia) and participation in other studies at the present time.

We randomly selected 24,862 patients from the study practices and checked them for multimorbidity and exclusion criteria. 13,935 of these patients were not multimorbid according to our definition or suffered from dementia. 3755 patients were excluded because of the other exclusion criteria described above. The remaining 7172 patients were eligible for study participation and contacted for informed consent to participation in our study. 3317 patients agreed to participate which corresponds to a total response rate of 46.2 %. Retrospectively we had to exclude 128 patients, because they died before the baseline interview or we found out in contact with the patients that they complied with the exclusion criteria without the GP’s knowledge. After all, 3189 patients could be included in the study [15]. Recruitment and data collection took place from July 2008 to October 2009.

### Measures

We used the patients’ morbidity data from standardized GP interviews at baseline based on a list of 46 groups of chronic conditions. This list was designed to cover the most prevalent chronic conditions from both primary care and ambulatory insurance claims data. The methods for compiling the list of 46 diagnosis groups have been described elsewhere in detail [16]. In short, we used the most frequent conditions in GP surgeries as mentioned in a panel survey of the Central Research Institute of Statutory Ambulatory Health Care in Germany (“ADT-Panel”) [17]. Chronicity of diagnoses was assessed using the scientific expert report for the formation of a morbidity orientated risk adjustment scheme in the German Statutory Health Insurance [18]. In order to capture a comprehensive picture of the disease patterns in individual patients we amended this list for all chronic conditions with a prevalence ≥ 1 % in the age group ≥ 65 years in the data set of the Germany statutory health insurance company Gmünder ErsatzKasse in 2006.

We also included the patients’ age and gender from GP charts. Chronic pain was operationalized as self-reported pain intensity (in matters of worst pain, on average and at the present time) and pain-related disability (i.e., pain interfering with recreational, social and family activities and the ability to work) persisting over the last six months and assessed in comprehensive patient interviews using the Graded Chronic Pain Scale (GCPS) [19]. For the analyses presented here the GCPS summary score was grouped into two categories: 1) “without chronic pain” consisting of the GCPS categories “pain free” and “low disability-low intensity” and 2) “with chronic pain” consisting of the GCPS categories “low disability-high intensity”, “high disability-moderately limiting” and “high disability-severely limiting”. We decided to include patients with low pain intensity/low disability into the group “without chronic pain”, because otherwise 85 % of the female patients would have been classified as “with chronic pain”. The patient interviews also included a number of other measuring instruments [4], which were not used for this paper.

Missing values in the dataset arising from item non-response have been imputed by Hot Deck Imputation in order to avoid bias generated by listwise deletion of subjects with missing values from statistical analyses [15]. Related to the analyses presented here 3134 patients (98.3 %) had complete data sets without any missing values. Age, gender and the 46 chronic conditions did not contain any missing values, but we imputed missing values in the summary score of the Graded Chronic Pain Scale (1.7 % missing values). Imputation was performed with R 2.13.0 and the R-package StatMatch 1.0.2.

### Analyses

Descriptive data were presented separately for both genders as means and standard deviations in case of continuous variables and as percentages in case of categorical variables. We excluded missing values from our descriptive analyses and reported the number of available data sets. Statistical significance of differences between genders was assessed by t-tests and *X*^2^-tests.

We used CART (classification and regression tree) analyses to identify diseases and diseases combinations bearing a higher proportion of patients with chronic pain than in the total study population. This method splits the data set recursively into two subsets based on the predictor variable (here: the chronic disease) which has the largest effect on the outcome variable (here: the proportion of patients with chronic pain). This process is repeated for each subset as long as the criteria for splitting are true. The 46 chronic conditions from our list were used as predictor variables and the dichotomized GCPS score was used as outcome variable. Splitting eligibility of predictor variables were determined by *X*^2^-tests. Bonferoni-adjustment for multiple testing was applied. A minimum sample size of 100 cases was defined for father branches and 50 cases for child branches. Stability of the branches was assured by 10-fold cross-validation. We performed our CART analyses for both genders separately, because there are big differences between males and females in the prevalence rates of pain symptoms and chronic conditions and we, therefore, wanted to allow for a possibly different association structure.

For all analyses an alpha-level of 5 % (i.e., p ≤ 0.05) was defined as statistically significant. Descriptive statistics, t-tests and *X*^2^-tests were conducted using Stata 12.1. CART analyses were performed using SPSS 20.0.0.

## Results

About 60 % of the 3189 multimorbid study participants were female (cf. Table 1). Females had a slightly higher age than males. There was a much higher percentage of people with high pain intensity and high pain-related disability among women than among men. In contrast the proportion of pain free individuals was nearly twice as high among males than among females. The mean number of chronic conditions per patient (from our list of 46 entities) approximately was the same between both genders, but women significantly more often suffered from chronic low back problems, joint arthrosis and depression while men were more likely to be affected from chronic ischemic heart disease, hyperuricemia/gout, neuropathies and cardiac insufficiency.

**Table 1:** Age, pain level and selected chronic conditions by gender

	Females *n* = 1891 (59.3 %)	Males *n* = 1298 (40.7 %)	*p*
Age: mean ± sd	74.7 ± 5.3 years	74.0 ± 5.1 years	< 0.001
Graded Chronic Pain Scale: summary score			< 0.001
Pain free	15.3 %	29.6 %	
Low disability - low intensity	40.4 %	41.8 %	
Low disability - high intensity	21.6 %	13.2 %	
High disability - moderately limiting	12.4 %	9.4 %	
High disability - severely limiting	10.4 % (*n* = 1860)	6.0 % (*n* = 1274)	
Number of chronic condition^a^: mean ± sd	7.0 ± 2.4	7.1 ± 2.5	0.068
Prevalence of chronic conditions			
Chronic low back pain	55.2 %	41.1 %	< 0.001
Joint arthrosis	48.9 %	35.3 %	< 0.001
Chronic ischemic heart disease	22.2 %	44.7 %	< 0.001
Depression	22.6 %	10.6 %	< 0.001
Hyperuricemia/Gout	12.9 %	23.7 %	< 0.001
Neuropathies	13.0 %	17.3 %	0.001
Intestinal diverticulosis	15.5 %	13.0 %	0.051
Cardiac insufficiency	11.8 %	15.0 %	0.009
Chronic gastritis/Gastroesophageal reflux	13.6 %	11.9 %	0.153

*sd* standard deviation; ^a^ out of a list of 46 chronic conditions

**Fig. 1:**
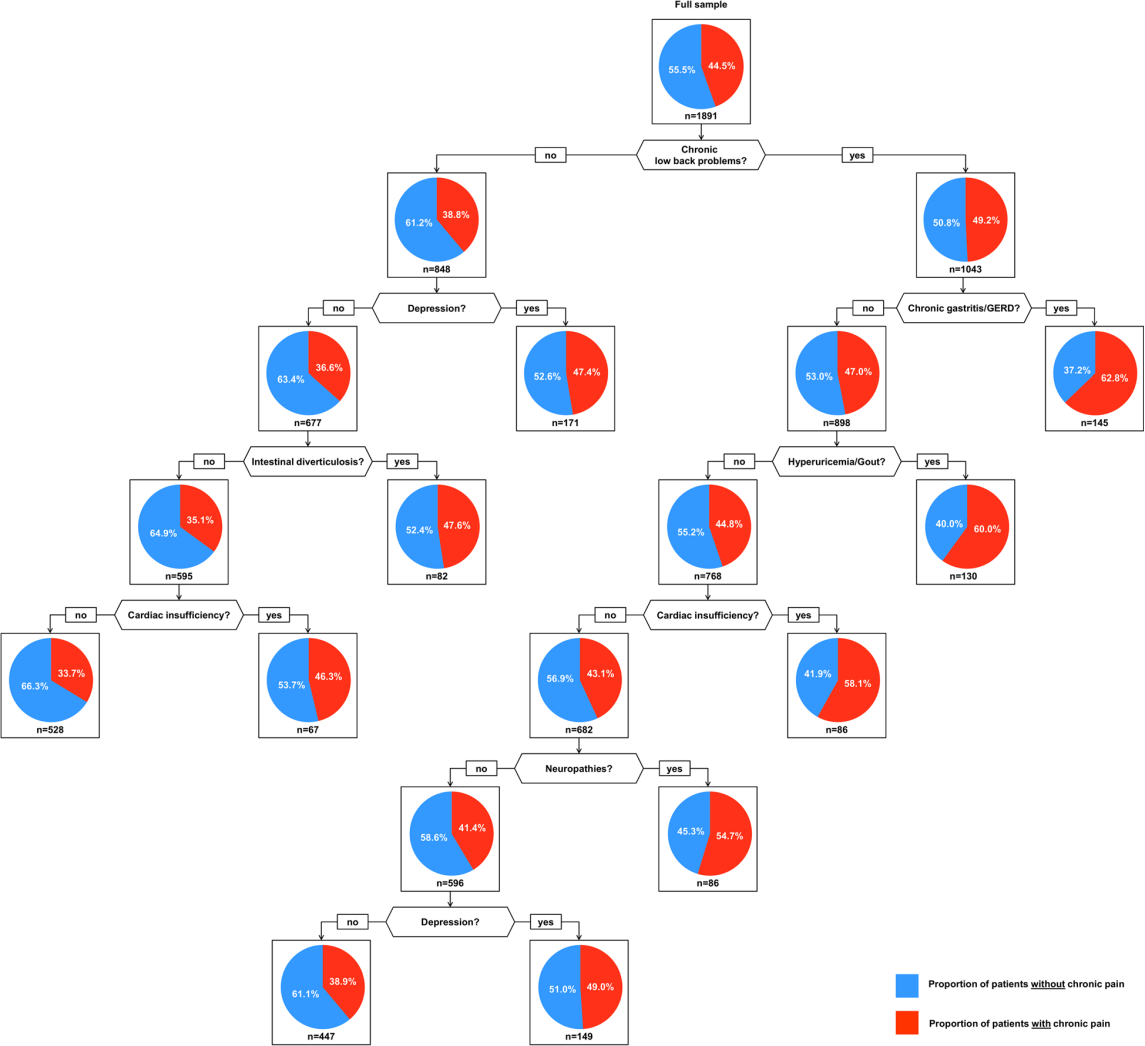
The association between disease combinations and pain in women. GERD: Gastroesophageal reflux

**Fig. 2:**
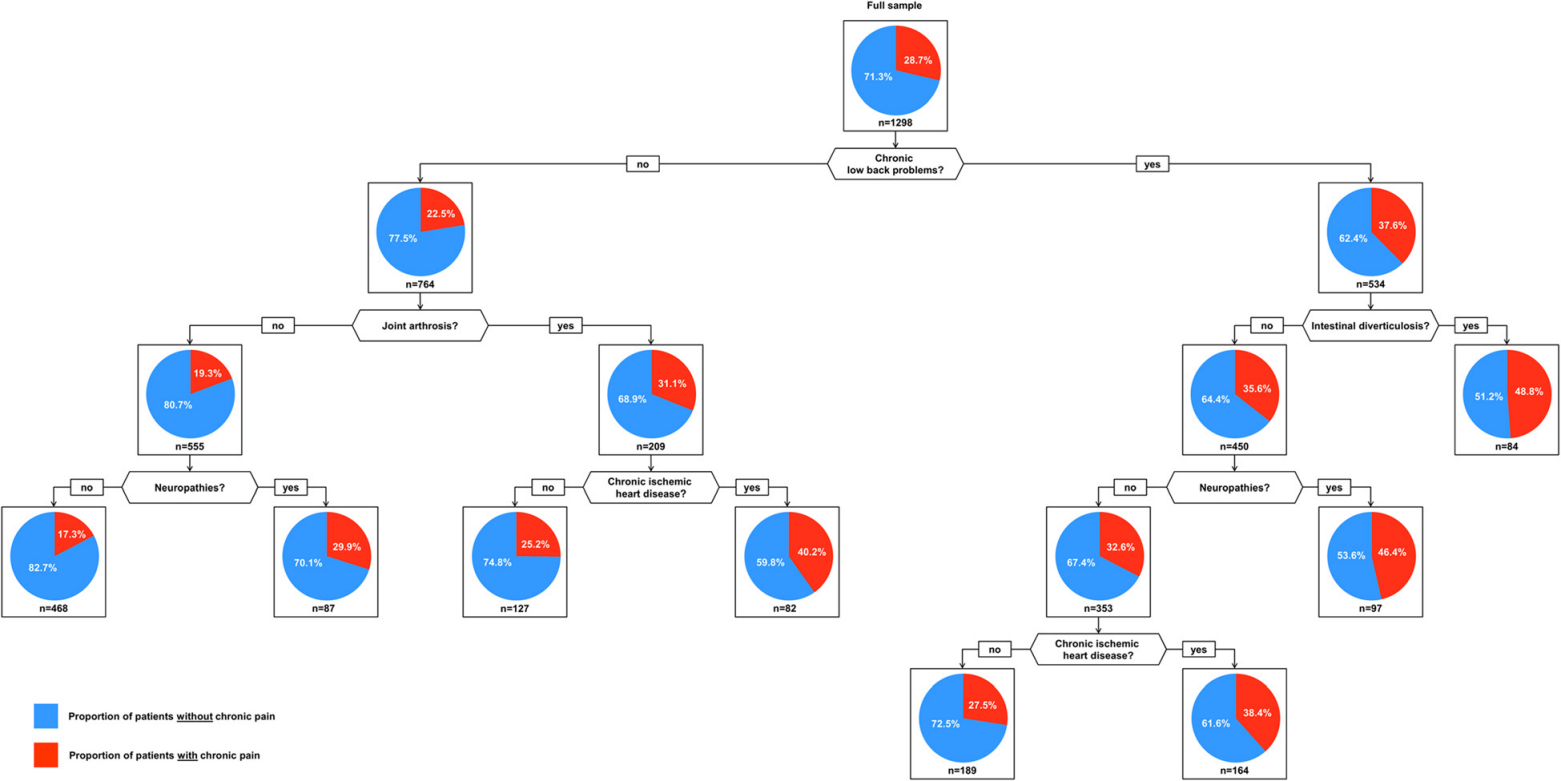
The association between disease combinations and pain in men

The association between disease combinations and pain is shown in Fig. 1 for women and Fig. 2 for men. According to our definition 44.5 % of the female population suffered from chronic pain. The most important chronic condition in the female population regarding the pain level was chronic low back problems, which led to higher proportion of chronic pain (49.2 %), especially if it was combined with chronic gastritis/gastroesophageal reflux disease (62.8 %), hyperuricemia/gout (60.0 %), cardiac insufficiency (58.1 %), neuropathies (54.7 %) or depression (49.0 %). On the other hand, female patients were less likely to suffer from pain if they were not affected by chronic low back problems (38.8 %) and also not by depression (36.6 %), intestinal diverticulosis (35.1 %) and cardiac insufficiency (33.7 %). The proportion of cases incorrectly classified in women was 40.0 %.

Among male patients only 28.7 % suffered from pain. Related to chronic pain the male population was also divided best by chronic low back problems, which was associated with a higher likelihood of pain (37.6 %), especially if combined with intestinal diverticulosis (48.8 %), neuropathies (46.4 %) or chronic ischemic heart disease (38.4 %). Among male patients without chronic low back problems, the proportion of individuals with pain was lower (22.5 %), especially if they also did not suffer from joint arthrosis (19.3 %) and neuropathies (17.3 %). Compared to the general (multimorbid) male population men without back problems, but with joint arthrosis also had an increased probability of pain (31.1 %), especially if they had the additional diagnosis of chronic ischemic heart disease (40.2 %). The proportion of cases incorrectly classified in men was 28.7 %.

## Discussion

### Main results

In this cross sectional analysis of the MultiCare Cohort Study chronic low back problems emerged as the most important chronic condition dividing our population of multimorbid primary care patients in chronic pain sufferers and non-chronic pain sufferers. In our data set 79 % of back problem diagnoses in females and 83 % in males were spondylosis, intervertebral disc disorders, spinal osteochondrosis or other degenerative back problems. Although back problems seem to be the condition with the strongest association with chronic pain, more than 50 % of women and more than 60 % of men with the diagnosis of chronic low back problems do not report chronic pain symptoms. For these reasons the diagnosis group “chronic low back problems” seems to incorporate mostly organic complaints which are or are not related to chronic pain.

In both, women and men, chronic low back problems are located in the top of a CART-tree of comorbid disease clusters and both trees include neuropathies and intestinal diverticulosis, however, the other elements of these clusters differ between genders. While the role of depression, cardiac insufficiency, hyperuricemia/gout and chronic gastritis/GERD (gastroesophageal reflux disease) is stressed in the female population, chronic pain in the male population more depended on chronic ischemic heart disease and joint arthrosis. Except for depression, which seems to be a larger problem in females than in males [20], there are no obvious explanations for these gender differences.

With regard to the pain clusters identified in both genders there is little agreement with our earlier analyses of disease-based multimorbidity clusters, which divided the disease spectrum in multimorbid patients into cardiovascular/metabolic, psychiatric/pain-related and neuropsychiatric disorders [9]. Chronic pain is related to chronic low back problems, joint arthrosis, depression, intestinal diverticulosis and chronic gastritis/GERD from the psychiatric/pain-related cluster, but also with chronic ischemic heart disease, cardiac insufficiency, hyperuricemia/gout and neuropathies from the cardiovascular/metabolic cluster. It therefore seems to be largely independent from disease-based clustering mechanisms in the multimorbid population.

### Limitations

On the one hand, the misclassification rate was comparably high, especially in women. The reason for this finding is that our diseases have a low specificity in predicting chronic pain as chronic pain can have a huge variety of reasons and only the most prevalent are covered in our analyses. On the other hand there is a large spread between the disease combinations with the highest and the lowest proportion of chronic pain, which is 33.7 to 62.8 % in females and 17.3 to 48.8 % in males. Our CART trees therefore are not suitable for predicting chronic pain, but they are a first step in identifying disease combinations that are related to chronic pain for a high proportion of patients.

In terms of generalizability of the results, it should be considered that the MultiCare Cohort Study only includes elderly multimorbid patients from general practice. However, this group comprises 44 % of the patients in this age group [15]. We were able to obtain a participation rate of 46 %. Although this rate is similar to other studies with a comparable design [21], we cannot rule out a selection bias due to non-response. A non-responder analysis revealed that younger patients and patients with intestinal diverticulosis or psoriasis had a better chance of study participation. However, there was no selection bias due to gender and the other 27 diseases used for patient inclusion [15].

The generalizability of the MultiCare Cohort Study could also be affected by our criteria for exclusion at baseline. We excluded patients with dementia because of their inability to consent as well as patients residing in a nursing home. Our recruitment only took place in larger German cities, so that rural areas were not included in our study. Nevertheless, our study is representative of an older, urban, multimorbid cohort in primary care [15]. Like other observational studies chronic pain was obtained by self-report. Prevalences of chronic pain surveys vary according to the individual self-observation and reception of bodily complaints. As chronic diseases were assessed by GP interviews we do not have problems with common method variance in our results.

A strength of our study relates to a high data quality that results from the fact that interviewers were regularly trained and monitored and a multitude of procedures for prevention of insufficient data quality, detection of inaccurate or incomplete data and actions to improve data quality were performed, e.g., user reliability trainings, automatic plausibility and integrity checks and data error reports to the collaborating centres.

There are also some limitations that result from the CART method. Although we conducted separate analyses for both genders, we could not adjust for other possibly confounding factors. For this reason our results might be confounded by variables like age or socio-economic status if they are associated with the prevalence of specific chronic conditions and also have an influence on the prevalence of chronic pain. Furthermore CART-analyses divide the data set by the best separating variables until the subsets are too small to continue (in terms of sample size and statistical significance of differences between child branches). As the sample size rapidly decreases with each split we might have missed some diseases or disease combinations associated with a higher prevalence of chronic pain.

### Comparison with literature

As reported in the literature, chronification of low back pain is associated with decreased subjective general health, decreased socioeconomic status, stress and psychosocial co-symptoms as indicated below [22]. In a prospective study Meyer et al. reported that depression is not either a risk factor or a consequence of disabling low back pain in community-dwelling elderly but that depressive symptoms are intermingled with the course of disabling low back pain in this group [23]. Meyer et al. could also demonstrate that an amplification model on chronic back pain, including amplification to psychological distress, is related to outcome of medical rehabilitation [24].

As suggested above, low back problems have an important role in patients with multimorbidity. Low back pain is one of the most frequent consultation reasons in primary care [25, 26]. Both, low back problems as well as multimorbidity are burdensome with regard to medical as well as financial aspects. Low back problems themselves are interrelated with a large variety of comorbidities [27]. In their retrospective analysis of measures of comorbidities in low back problems, Ritzwoller et al. [28] identified physical and mental health co-morbidities and measures of analgesic use to be associated with chronicity, healthcare utilization and costs. In their study, the prevalence of comorbidities varied with number of low back pain episodes. Diabetes, rheumatoid arthritis, anxiety, psychotic illness, depression, use of opiates and NSAIDs were associated with significant incremental increases in costs.

Also, in chronification of pain derived from other body regions psychosocial factors are suggested to have a higher impact than biomedical (i.e., “somatic”) issues. Therefore, anxiety and depression as well as somatoform disorders and dysfunctional coping strategies have previously been described as the main drivers of pain chronification [29]. In line with Beyer and Steinberger and also the above mentioned study of Meyer, depression played an important role in terms of association with chronic pain in our female patients. However, since psychosocial and emotional problems are suggested to influence both, chronification of low back pain as well as chronification of pain in general, these might be an important issue to be kept in mind by GPs and other professionals dealing with multimorbidity.

## Conclusions

Our explorative analyses are a first step in identifying diseases and disease combinations that are related to chronic pain for a high proportion of patients. The most important condition in multimorbid primary care patients suffering from chronic pain seems to be chronic low back problems. In both genders, chronic low back pain as well as chronic pain in other body regions seem to be strongly interrelated with cardiometabolic conditions. In women, psychosocial issues like depression also seem to be relevant. However, more research is needed to better understand the patient perspective regarding pain symptoms associated with multiple chronic conditions.

Our results have a number of implications. In medical education, information about the relationship between chronic pain problems and comorbidity should be given. And in health care for patients with multimorbidity, possible pain symptoms resulting from chronic low back problems should be discussed when prioritizing diseases. Furthermore, interactions of back pain with cardiometabolic and psychiatric disorders should be assessed in order to prevent chronification of pain, if possible.

## Abbreviations

CART, classification and regression tree; GCPS, Graded Chronic Pain Scale; GERD, gastroesophageal reflux disease; GP, general practitioner.

## References

[1] Marengoni A, Angleman S, Melis R, Mangialasche F, Karp A, Garmen A, Meinow B, Fratiglioni L. Aging with multimorbidity: a systematic review of the literature. Ageing Res Rev. 2011;10:430–9. 10.1016/j.arr.2011.03.003 21402176

[2] Fortin M, Stewart M, Poitras M-E, Almirall J, Maddocks H (2012). A systematic review of prevalence studies on multimorbidity: toward a more uniform methodology. Ann Fam Med.

[3] Van den Akker M, Buntinx F, Knottnerus JA (1996). Comorbidity or multimorbidity: what’s in a name? A review of literature. Eur J Gen Pract.

[4] Schäfer I, Hansen H, Schön G, Maier W, Höfels S, Altiner A, Fuchs A, Gerlach FM, Petersen JJ, Gensichen J, Schulz S, Riedel-Heller S, Luppa M, Weyerer S, Werle J, Bickel H, Barth K, König HH, Rudolph A, Wiese B, Prokein J, Bullinger M, von dem Knesebeck O, Eisele M, Kaduszkiewicz H, Wegscheider K, van den Bussche H. The German MultiCare-study: patterns of multimorbidity in primary health care - protocol of a prospective cohort study. BMC Health Serv Res. 2009;9:145. 10.1186/1472-6963-9-145 PMC3224741 19671164

[5] Tinetti ME, Fried T (2004). The end of the disease era. Am J Med.

[6] Starfield B (2006). Threads and yarns: weaving the tapestry of comorbidity. Ann Fam Med.

[7] Wiesner G, Grimm J, Bittner E (2003). [Multimorbidity in Germany. State – Development – Consequences].

[8] Gijsen R, Hoeymans N, Schellevis FG, Ruwaard D, van den Satariano WA, Bos GA (2001). Causes and consequences of comorbidity: a review. J Clin Epidemiol.

[9] Schäfer I, von Leitner EC, Schön G, Koller D, Hansen H, Kolonko T, Kaduszkiewicz H, Wegscheider K, Glaeske G, van den Bussche H. Multimorbidity patterns in the elderly: a new approach of disease clustering identifies complex interrelations between chronic conditions. PLoS One. 2010;5:e15941. 10.1371/journal.pone.0015941 PMC3012106 21209965

[10] Wolff R, Clar C, Lerch C, Kleijnen J (2011). Epidemiology of chronic non-malignant pain in Germany. Schmerz.

[11] Butchart A, Kerr EA, Heisler M, Piette JD, Krein SL (2009). Experience and management of chronic pain among patients with other complex chronic conditions. Clin J Pain.

[12] Freytag A, Quinzler R, Freitag M, Bickel H, Fuchs A, Hansen H, Hoefels S, König HH,·Mergenthal·K, Riedel-Heller SG,·Schön G, Weyerer S, Wegscheider K, Scherer M, van den Bussche H, Haefeli WE, Gensichen J. [Use and potential risks of over-the-counter analgesics]. Schmerz. 2014;28:175–82. 10.1007/s00482-014-1415-5 24718747

[13] Blozik E, van den Bussche H, Gurtner F, Schäfer I, Scherer M (2013). Epidemiological strategies for adapting clinical practice guidelines to the needs of multimorbid patients. BMC Health Serv Res.

[14] Gloth FM (2011). Pharmacological management of persistent pain in older persons: focus on opioids and nonopioids. J Pain.

[15] Schäfer I, Hansen H, Schön G, Höfels S, Altiner A, Dahlhaus A, Gensichen J, Riedel-Heller S, Weyerer S, Blank W, König HH, von dem Knesebeck O, Wegscheider K, Scherer M, van den Bussche H, Wiese B. The influence of age, gender and socio-economic status on multimorbidity patterns in primary care. First results from the MultiCare Cohort Study. BMC Health Serv Res. 2012;12:89. 10.1186/1472-6963-12-89 PMC3348059 22471952

[16] Van den Bussche H, Koller D, Kolonko T, Hansen H, Wegscheider K, Glaeske G, von Leitner EC, Schäfer I, Schön G. Which chronic diseases and disease combinations are specific to multimorbidity in the elderly? Results of a claims data based cross-sectional study in Germany. BMC Public Health. 2011;11:101. 10.1186/1471-2458-11-101 PMC3050745 21320345

[17] Zentralinstitut für die kassenärztliche Versorgung: ADT-Panel. http://www.zi.de/cms/projekte/adt-panel. 2007. Accessed 17 March 2016.

[18] Busse R, Drösler S, Glaeske G, Greiner W, Schäfer T, Schrappe M (2007). Scientific opinion for the selection of 50 to 80 diseases for consideration in the morbidity-oriented risk adjustment.

[19] Von Korff M, Ormel J, Keefe FJ, Dworkin SF (1992). Grading the severity of chronic pain. Pain.

[20] Piccinelli M, Wilkinson G (2000). Gender differences in depression. Critical review. Brit J Psychiat.

[21] Luck T, Riedel-Heller SG, Kaduszkiewicz H, Bickel H, Jessen F, Pentzek M, Wiese B, Kölsch H, van den Bussche H, Abholz HH, Mösch E, Gorfer S, Angermeyer MC, Maier W, Weyerer S. Mild cognitive impairment in general practice: age-specific prevalence and correlates results from the German Study on Ageing, Cognition and Dementia in Primary Care Patients (AgeCoDe). Dement Geriatr Cogn Disord. 2007;24:307–16. 10.1159/000108099 17848793

[22] Arnold B, Brinkschmidt T, Casser HR, Gralow I, Irnich D, Klimczyk K, Müller G, Nagel B, Pfingsten M, Schiltenwolf M, Sittl R, Söllner W. Multimodal pain therapy. Concepts and indication. Schmerz. 2009;23:112–20. 10.1007/s00482-008-0741-x 19156448

[23] Meyer T, Cooper J, Raspe H (2007). Disabling low back pain and depression in the community-dwelling elderly: a prospective study. Spine.

[24] Meyer T, Deck R, Hüppe A, Raspe H (2006). Amplification model on chronicity of back pain: analysis of discriminant and prognostic validity in rehabilitation inpatients. DRV-Schriften.

[25] Hart LC, Deyo RA, Cherkin DC (1995). Physician office visits for low back pain. Spine.

[26] Cassidy JD, Carroll LJ, Cote P (1998). The Saskatchewan health and back pain survey. Spine.

[27] Schäfer I, Kaduszkiewicz H, Wagner HO, Schön G, Scherer M, van den Bussche H (2014). Reducing complexity: a visualisation of multimorbidity by combining disease clusters and triads. BMC Public Health.

[28] Ritzwoller DP, Crounse L, Shetterly S, Rublee D (2006). The association of comorbidities, utilization and costs for patients identified with low back pain. BMC Musculoskelet Disord.

[29] Beyer A, Steinberger M (2005). Chronic pain syndroms in non-cancer patients. Dtsch Med Wochenschr.

